# Aqueous *Oldenlandia diffusa* extracts inhibits colorectal cancer cells via activating AMP-activated protein kinase signalings

**DOI:** 10.18632/oncotarget.9969

**Published:** 2016-06-13

**Authors:** Pei-Hua Lu, Min-Bin Chen, Chao Ji, Wen-Ting Li, Mu-Xin Wei, Mian-Hua Wu

**Affiliations:** ^1^ Jiangsu Collaborative Innovation Center of Traditional Chinese Medicine (TCM) Prevention and Treatment of Tumor, Nanjing University of Chinese Medicine, Nanjing, 210023, China; ^2^ Department of Medical Oncology, Wuxi People's Hospital Affiliated to Nanjing Medical University, Wuxi, 214023, Jiangsu, China; ^3^ Department of Medical Oncology, Kunshan First People's Hospital Affiliated to Jiangsu University, Kunshan, 215300, Jiangsu, China; ^4^ Department of Dermatology, The First Affiliated Hospital of Fujian Medical University, Fuzhou, 350005, Fujian, China; ^5^ Department of Traditional Chinese Medicine, First Affiliated Hospital of Nanjing Medical University, Nanjing, 210029, China

**Keywords:** colorectal cancer (CRC), Oldenlandia diffusa (OD) extracts (ODE), AMP-activated protein kinase (AMPK), p53 and mammalian target of rapamycin (mTOR)

## Abstract

Here we evaluated the anti-cancer activity of aqueous *Oldenlandia diffusa* (OD) extracts (ODE) in colorectal cancer (CRC) cells. We showed that ODE exerted potent anti-proliferative, cytotoxic and pro-apoptotic activities against a panel of established CRC lines (HCT-116, DLD-1, HT-29 and Lovo) and primary (patient-derived) human CRC cells. ODE activated AMP-activated protein kinase (AMPK) signaling, which led to subsequent mTORC1 inhibition and Bcl-2/HIF-1α downregulation in CRC cells. In ODE-treated CRC cells, AMPKα1 formed a complex with p53. This might be important for p53 activation and subsequent cancer cell apoptosis. Inhibition of AMPK signaling, though dominant negative (dn) mutation or shRNA/siRNA knockdown of AMPKα1 attenuated ODE-exerted CRC cytotoxicity. *In vivo*, i.p. administration of ODE inhibited HCT-116 xenograft tumor growth in SCID mice. In addition, AMPK activation, mTORC1 inhibition and p53 activation were observed in ODE-treated HCT-116 xenograft tumors. These results suggest that ODE inhibits CRC cells *in vitro* and *in vivo*, possibly via activation of AMPK-dependent signalings.

## INTRODUCTION

The application of conventional chemotherapy is limited in colorectal cancer (CRC) cells with pre-existing and/or acquired resistances [1]. In addition, our groups [2–4] and others have shown that molecular heterogeneity of CRCs hinders the uniform application of specific molecularly-targeted agents [5–7]. Therefore, studies are exploring novel and more efficient anti-CRC agents [8].

*Oldenlandia diffusa* (OD), a member of the *Rubiaceae* family, is a well-known medicinal plant in ancient China [9]. Existing evidences have described multiple biological functions of OD components, including anti-angiogenic, anti-inflammatory, anti-oxidant, and pro-apoptotic activities [9, 10]. More importantly, (OD) extracts (ODE) have displayed significant anti-cancer activity in a number of preclinical cancer studies [10–13]. However, the potential effect of ODE in CRC cells has not been extensively studied.

Our studies [14, 15] have implied that AMP-activated protein kinase (AMPK), the master energy sensor, is also an important mediator of cell death and apoptosis under various stress conditions (see review [16]). In multiple cancer cell lines, various anti-cancer agents and natural occurring compounds were shown to activate AMPK-dependent cell apoptosis/death pathways [14, 16–26]. In the current study, we show that ODE potently inhibits CRC cells *in vitro* and *in vivo*. Activation of AMPK could be the major signaling mechanisms responsible for ODE's actions in CRC cells.

## RESULTS

### *Oldenlandia diffusa* extracts (ODE) inhibits CRC cell proliferation and survival

**Figure 1:**
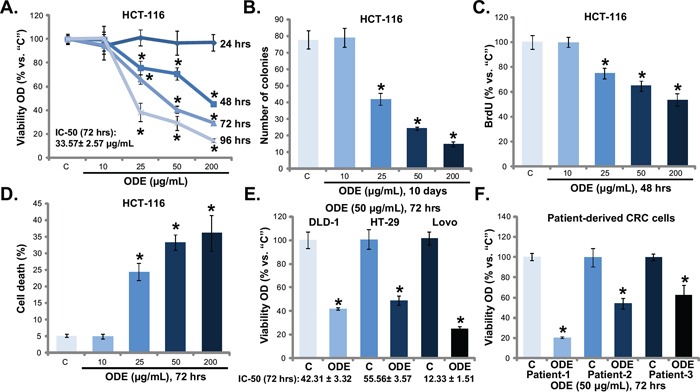
*Oldenlandia diffusa* extracts (ODE) inhibits CRC cell proliferation and survival A panel of established CRC cell lines (HCT-116, Lovo, HT-29 and DLD-1) or three primary human CRC cell lines were treated with or without ODE at applied concentrations, cells were further cultured, and cell proliferation was evaluated by MTT assay **A**, **E** and **F**., colony formation assay (**B.**, for HCT-116 cells) and BrdU incorporation assay (**C**., for HCT-116); Cell death was analyzed by the trypan staining assay (**D.**, for HCT-116). “C” stands for untreated control group (Same for all Figures). For each assay, n=5 (Same for all Figures). Data in this figure were repeated four times, and similar results were obtained. * *p* < 0.05 vs. “C” group.

MTT assay results in Figure 1A showed that ODE inhibited HCT-116 cell proliferation (MTT viability reduction). The anti-proliferative activity by ODE in HCT-116 cells was concentration- and time-dependent (Figure 1A). The colony formation assay results in Figure 1B and BrdU incorporation assay in Figure 1C further confirmed the anti-proliferative activity of ODE when applied in HCT-116 cells. The number of proliferative HCT-116 colonies (Figure 1B) and BrdU incorporation (Figure 1C) were both dramatically decreased after ODE (25-200 μg/mL) treatment. A low-concentration of ODE (10 μg/mL) showed no significant effect on HCT-116 cell proliferation (Figure 1B and 1C, *p* > 0.05 *vs.* control group). Trypan blue staining assay results in Figure 1D demonstrated that ODE at 25-200 μg/mL induced significant HCT-116 cell death.

Next, we studied the potential activity of ODE to other human CRC cells. MTT results in Figure 1E showed that ODE (50 μg/mL) inhibited the proliferation of three other established CRC cell lines, including DLD-1, HT-29 and Lovo. We also calculated the IC-50 of ODE in above CRC cells with different p53 status. The IC-50 of ODE was low in p53-wild HCT-116 (33.57± 2.57 μg/mL) and LoVo (12.33 ± 1.51 μg/mL) CRC cells [33–35], but was relatively high in p53-mutant HT-29 (55.56± 3.57μg/mL) and DLD-1 (42.31 ± 3.32μg/mL) cells [33–35]. Meanwhile, we established three lines of patient-derived primary CRC cells based on the method described [2]. These primary CRC cells were also incubated with ODE-containing medium. MTT assay was again performed, and results (Figure 1F) showed that ODE (50 μg/mL) inhibited proliferation of all three lines of primary CRC cells. Together, these results show that ODE exerts potent anti-proliferative and cytotoxic activity against human CRC cells.

### ODE activates apoptosis in CRC cells

**Figure 2:**
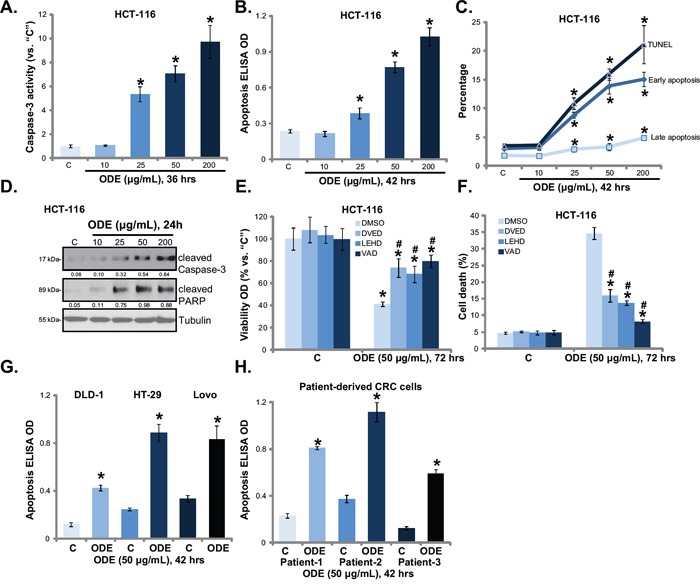
ODE activates apoptosis in CRC cells A panel of established CRC cell lines (HCT-116, Lovo, HT-29 and DLD-1) and three primary human CRC cell lines were treated with or without ODE at applied concentrations, cells were further cultured, cell apoptosis was analyzed by listed assay **A**-**D**, **G** and **H**. HCT-116 cells were pretreated with Ac-DEVD-CHO (“DVED”), Ac-LEHD-CHO (“LEHD”) or Ac-VAD-CHO (“VAD”) (40 μM each) for 1 h, following by ODE (50 μg/mL) treatment, cell viability **E**. and cell death **F**. were tested. “DMSO” stands for 0.1% DMSO. Cleaved-PARP/cleaved-caspase-3 expression (vs. Tubulin) was quantified. Data in this figure were repeated four times, and similar results were obtained. * *p* < 0.05 vs. “C” group. ^#^ *p* < 0.05 vs. “ODE” only group (E and F).

Next, several apoptosis assays were performed to test cell apoptosis in ODE-treated CRC cells. Results demonstrated that ODE (25-200 μg/mL) induced significant apoptosis activation in HCT-116 cells. The caspase-3 activity (Figure 2A), Histone DNA ELISA OD (Figure 2B), the percentage of Annexin V or TUNEL positive cells (Figure 2C) were all increased following ODE (25-200 μg/mL) treatment in HCT-116 cells. Meanwhile, the expressions of cleaved-poly (ADP-ribose) polymerase (PARP) and cleaved-caspase-3 were increased in ODE (25-200 μg/mL)-treated HCT-116 cells (Figure 2D). Once again, the low-concentration of ODE (10 μg/mL) showed no significant effect on HCT-116 cell apoptosis (Figure 2A-2D).

Next, the apoptosis inhibitors, including the caspase-3 specific inhibitor Ac-DEVD-CHO, the caspae-9 specific inhibitor Ac-LEHD-CHO and the caspae-8 specific inhibitor Ac-ITED-CHO, were applied. MTT assay results in Figure 2E and trypan blue staining assay results in Figure 2F showed that the three caspase inhibitors significantly attenuated ODE (50 μg/mL)-induced anti-proliferative and cytotoxic actions against HCT-116 cells. These results suggest that caspase-dependent apoptosis activation mediates ODE-induced anti-CRC cell activity. Further, ODE (50 μg/mL) was again pro-apoptotic in other established (DLD-1, HT-29 and Lovo) (Figure 2G) and primary (Figure 2H) human CRC cells. Note that apoptosis was tested by histone-DNA ELISA assay (Figure 2G and 2H). The above caspase inhibitors similarly alleviated ODE-mediated cytotoxicity against above established (DLD-1, HT-29 and Lovo) and primary CRC cells (Data not shown). Collectively, these results show that ODE induces apoptotic death in cultured CRC cells.

### ODE activates AMPK signaling in CRC cells

**Figure 3:**
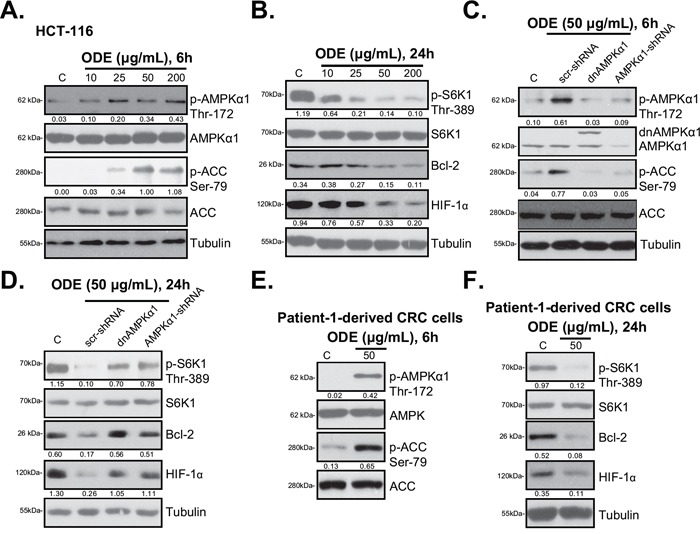
ODE activates AMPK signaling in CRC cells HCT-116 cells or patient-1-derived primary CRC cells were treated with or without applied ODE, cells were further cultured, expressions of listed proteins were tested by Western blots **A**, **B**, **E** and **F**. Stable HCT-116 cells expressing scramble control shRNA (“scr-shRNA”), AMPKα1-shRNA or dominant negative (dn)-AMPKα1 (“dnAMPKα1”) were treated with or without applied ODE, cells were further cultured for 6 h **C.** or 24 h **D**., expressions of listed proteins were tested by Western blots. Kinase phosphorylations and Bcl-2/HIF-1α expressions were quantified. Data in this figure were repeated three times, and similar results were obtained.

Existing evidences implied that AMPK activation inhibits cancer cells through regulating its downstream signals, including activating pro-apoptotic p53 cascades, and inactivating pro-cancerous mTOR complex 1 (mTORC1) signaling [16]. As shown in Figure 3A, ODE treatment in HCT-116 cells induced significant AMPK signaling activation, evidenced by AMPKα1/ACC phosphorylations [29]. Activation of mTORC1 (p-S6K1), and expressions of mTORC1-dependent genes (Bcl-2 [28, 29] and HIF-1α [36, 37]) were remarkably inhibited by ODE treatment in HCT-116 cells (Figure 3B). Inactivation of AMPK, via AMPKα1 shRNA (Figure 3C) [29] or dominant negative (dn) mutation (T172A, Figure 3C) [29], restored S6K1 phosphorylation (Figure 3D) and Bcl-2/HIF-1α expression in ODE-treated HCT-116 cells (Figure 3D). These results suggest that ODE activates AMPK signaling to inhibit mTOR activation (p-S6K1) and mTOR-regulated gene (Bcl-2 and HIF-1α) expression in HCT-116 cells. Similar results were also obtained in HT-29 cells and DLD-1 cells (Data not shown). In primary human CRC cells (patient-1 derived), ODE-treatment also activated AMPK signaling (AMPKα/ACC phosphorylations) (Figure 3E). p-S6K1 and Bcl-2/HIF-1α expressions were also inhibited (Figure 3F). Same results were seen in two other primary CRC cell lines (Data not shown). Thus, these results suggest that ODE activates AMPK to inhibit mTORC1 activation in CRC cells.

### AMPK activation mediates ODE-induced anti-CRC cell activity

**Figure 4:**
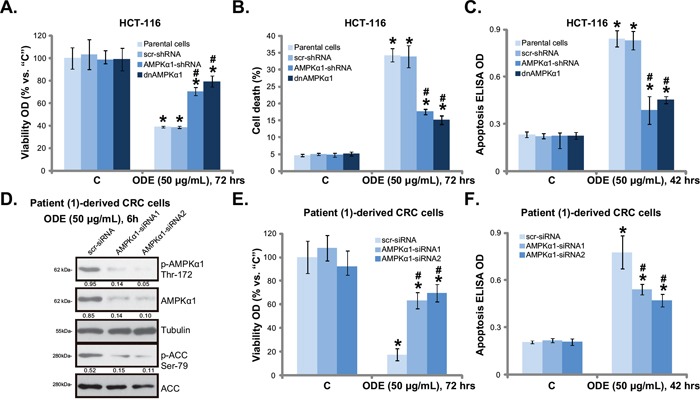
AMPK activation is required for ODE-induced anti-CRC cell activity Stable HCT-116 cells expressing scramble control shRNA (“scr-shRNA”), AMPKα1-shRNA or dominant negative (dn)-AMPKα1 (“dnAMPKα1”) as well as their parental cells were treated with or without applied ODE, cells were further cultured, cell viability (**A.**, MTT assay), cell death (**B.**, trypan blue staining assay) and cell apoptosis (**C.**, Histone DNA ELISA assay) were tested. Primary CRC cells (patient-1-dervied), transfected with scramble control siRNA (“scr-siRNA”) or AMPKα1-siRNA (−1/−2), were treated with ODE for indicated time, expressions of listed proteins were shown **D**., cell viability **E**. and apoptosis **F**. were tested similarly. Kinase phosphorylations were quantified (D). Data in this figure were repeated three times, and similar results were obtained. * *p* < 0.05 vs. “C” of “scr-shRNA”/“scr-siRNA” group. ^#^ *p* < 0.05 vs. “ODE” of “scr-shRNA”/“scr-siRNA” group.

Using the same genetic strategies, we showed that ODE-exerted HCT-116 cell viability reduction (Figure 4A), cell death (Figure 4B) and apoptosis (Figure 4C) were significantly attenuated with AMPKα1 silencing or mutation. Similar results were also obtained in HT-29 cells (Data not shown). Thus, we propose that ODE treatment in CRC cells induces a profound AMPK activation, causing mTORC1 in-activation, Bcl-2/HIF-1α downregulation, which might be responsible for CRC cell growth inhibition and apoptosis. In patient (−1)-derived primary CRC cells, siRNA strategy was utilized to transiently knockdown AMPKα1 in primary CRC cells. The two non-overlapping AMPKα1 siRNAs [32] both inhibited AMPKα1 expression and activation by ODE in primary cells (Figure 4D). As a consequence, ODE-exerted anti-proliferative (Figure 4E) and pro-apoptotic (Figure 4F) activities were attenuated in AMPKα1-silenced primary cancer cells. Similar results were also observed in two other primary cancer cell lines (Data not shown). Together, these results suggest that AMPK activation mediates ODE-induced anti-CRC cell activity.

### ODE activates p53 signaling in CRC cells

**Figure 5:**
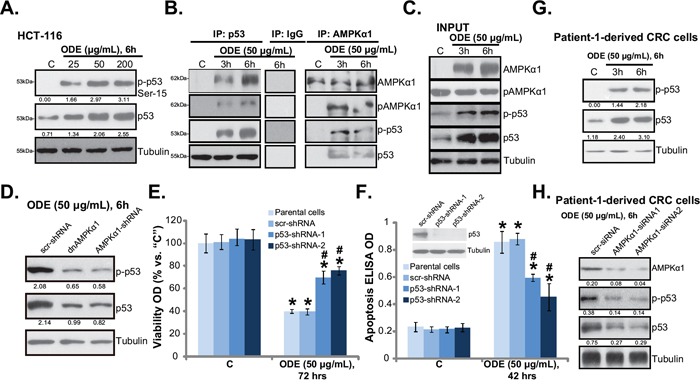
ODE activates p53 signaling in CRC cells HCT-116 cells were treated with or without ODE at applied concentrations, cells were further cultured, expressions of listed proteins were tested by Western blots **A** and **C**., the association between AMPKα1 (regular and p-) and p53 (regular and p-) was examined by co-immunoprecipitation (“Co-IP”) assay **B**., IgG was also included as a Co-IP control (B). Stable HCT-116 cells expressing scramble-shRNA (“scr-shRNA”), AMPKα1-shRNA or dominant negative (dn)-AMPKα1 (“dnAMPKα1”) were treated with applied ODE, p53 (regular and p-) and Tubulin expressions were tested by Western blots **D.** Stable HCT-116 cells expressing scramble-shRNA (“scr-shRNA”) or p53-shRNA (“−1/−-2”) as well as their parental cells were treated with applied ODE, cell viability (MTT assay, **E.**) and cell apoptosis (Histone DNA ELISA assay, **F.**) were tested, expression of p53 in these cells was also shown (F, upper panel). p53 (regular and p-) and Tubulin expressions in ODE (50 μg/mL)-treated primary CRC cells (patient-1 derived) were shown **G**. p53 (regular and p-) and AMPKα1 expressions in ODE (50 μg/mL)-treated primary CRC cells with scramble control siRNA (“scr-siRNA”) or AMPKα1 siRNA (“−1/−2”) were shown **H.** Kinase phosphorylations and p53 expression were quantified. Data in this figure were repeated three times, and similar results were obtained. * *p* < 0.05 vs. “C” of “scr-shRNA” group. ^#^ *p* < 0.05 vs. “ODE” of “scr-shRNA” group.

AMPK could activate p53-dependent apoptosis pathway in various cancer cells [15, 17, 29, 38, 39]. We showed that AMPK activation was required for vincristine-induced p53 activation and following melanoma cell apoptosis [14]. C6 ceramide and vincristine synergistically activated AMPK-p53 signaling to inhibit proliferation of multiple cancer cell lines [29]. Western blot results in Figure 5A showed that cytotoxic ODE (25-200 μg/mL) treatment in HCT-116 cells induced significant p53 activation, which was tested by p53 phosphorylation (at Ser-15) and upregulation (Figure 5A). Significantly, the co-immunoprecipitation (Co-IP) assay results in Figure 5B showed that ODE treatment induced AMPKα1 and p53 association in HCT-116 cells. More importantly, activated AMPKα1 (p-Thr-172) and activated p53 (p-Ser-15) formed a complex in ODE-treated HCT-116 cells (Figure 5B). The “INPUT” results in Figure 5C again confirmed AMPK and p53 activation by ODE in HCT-116 cells. The above results suggest a potential role of AMPK in ODE-induced p53 activation in CRC cells. To support this hypothesis, we once again utilized same genetic strategies. Remarkably, as shown in Figure 5C, AMPKα1 shRNA knockdown or dominant negative mutation dramatically inhibited ODE-induced p53 activation in HCT-116 cells, suggesting that AMPK might be important for p53 activation in ODE-treated cells.

To study the role of p53 in ODE-induced anti-CRC cell activity, we once again applied shRNA strategy to selectively and stably knockdown p53 in HCT-116 cells (See method in our previous study [29]). Results showed that ODE-induced anti-proliferation (MTT viability reduction, Figure 5E) and apoptosis (Figure 5F) were largely attenuated in p53-sileced stable HCT-116 cells. Note that we utilized two non-overlapping p53 shRNAs, each showed high efficiency in downregulating p53 expression (Figure 5F, upper panel) and inhibiting ODE's actions in HCT-116 cells (Figure 5E and 5F). In primary human CRC cells (Patient-1), ODE treatment also induced significant p53 activation (Ser-15 phosphorylation and upregulation) (Figure 5G). Such an effect was again inhibited by AMPKα1 siRNA (Figure 5H). Similar results were also seen in two other patient-derived CRC cell lines (Data not shown). Together, these results show that ODE activates AMPK-dependent p53 signaling to inhibit CRC cells.

### ODE inhibits HCT-116 xenograft growth in SCID mice

**Figure 6:**
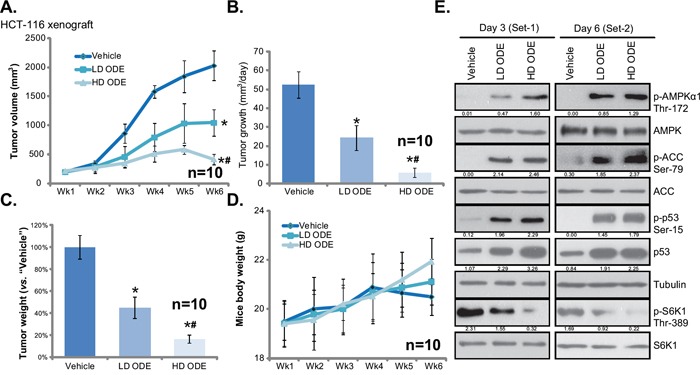
ODE inhibits HCT-116 xenograft growth in SCID mice Weekly HCT-116 xenograft tumor growth curve **A.** and mice body weight curve **D**. with indicated treatment: Saline (“Vehicle”), low-dose of ODE (0.2 g/kg, *i.p.*, daily, “LD ODE”), high-dose of ODE (1.0 g/kg, *i.p.*, daily, “HD ODE”), were shown (A). Tumor daily growth **B**. and tumor weights at week-6 **C**. were also presented. Three and six days after initial ODE administration, one mice per group were sacrificed, tumor tissues were removed and were subjected to Western blot assay of listed proteins **E**. Kinase phosphorylations and p53 expression were quantified (E). *In vivo* experiments were repeated twice, and similar results were obtained * *p* < 0.05 vs. “Vehicle” group. ^#^ *p* < 0.05 vs. “LD ODE” group.

The *in vivo* anti-CRC activity by ODE was also tested. As described, HCT-116 cells were injected into the SCID nude mice to create mice xenografts. These mice were subjected to ODE administration. Tumor growth curve results in Figure 6A showed that ODE administration significantly inhibited HCT-116 xenograft growth in SCID mice. The *in vivo* anti-HCT-116 activity of ODE was again dose-dependent, the high-dose ODE (“HD ODE”, 1.0 g/kg, *i.p.*, daily) was more potent than low-dose ODE (“LD ODE”, 0.2 g/kg, *i.p.*, daily) in suppressing HCT-116 xenografts (Figure 6A). Further, tumor daily growth was also significantly inhibited in ODE-treated mice (Figure 6B). Once again “HD ODE” group showed slower tumor daily growth than the “LD ODE” group (Figure 6B). In addition, the tumor weights at week-6 of ODE-treated groups were lower than that of vehicle (Saline) group (Figure 6C). These results suggest that ODE administration inhibited HCT-116 xenograft growth in SCID mice. We failed to detect any obvious deleterious effects in experimental mice. Mice body weights were not affected by the ODE regimens (Figure 6D), indicating the relative safety of the ODE treatments. Together, we show that ODE *i.p.* administration potently inhibits HCT-116 xenograft growth in SCID mice. Next, xenografted tumors were isolated. Western blot analyzing these tumor tissues demonstrated significant AMPK activation (AMPKα1/ACC phosphorylations), p53 activation (Ser-15 phosphorylation and upregulation) and mTORC1 in-activation (p-S6K1 inhibition) by ODE administration *in vivo* (Figure 6E). The *in vivo* activity of ODE on these signalings was again dose-dependent (see Figure 6E, quantification), and was observed in tumor tissues 3 days and 6 days after initial ODE administration (Figure 6E). Therefore, in line with *in vitro* findings, these results suggest that ODE administration results in AMPK-p53 activation and mTORC1 inhibition in xenografted HCT-116 tumors.

## DISCUSSIONS AND CONCLUSIONS

mTOR signaling is an important target for CRC intervention [40]. Our previous study has shown that INK-128, a novel mTOR kinase inhibitor, induced significant anti-tumor activity in preclinical CRC models [2]. AMPK is known to inhibit mTORC1 through at least two distinct mechanisms. First, AMPK phosphorylates and activates TSC2 (Tuberous sclerosis protein 2), a negative regulator of mTOR, to inactivate mTORC1. Second, AMPK direct phosphorylates and in-activates of Raptor (regulatory associated protein of mTOR), which is a key functional component of mTORC1 [41]. Here, we showed that ODE activated AMPK signaling to inhibit mTORC1 in CRC cells. S6K1 phosphorylation, the indicator of mTORC1 activation, as well expression of mTORC-1-regulated genes (Bcl-2 and HIF-1α) were all inhibited in ODE-treated CRC cells. Reversely, inhibition or silence of AMPK restored mTORC1 activation and Bcl-2/HIF-1α expression. These results suggest that ODE activates AMPK signaling to inhibit cancer-promoting mTORC1 signaling in CRC cells.

The role of p53 in cell apoptosis has been well-established. Recent studies have shown that ODE-induced anti-cancer activity could be through activating p53 signaling [10]. Thus, one important finding of this study is that AMPK is also important for p53 activation by ODE in CRC cells. Our results showed that activated AMPKα1 formed a complex with p53 in ODE-treated CRC cells, which might be important for subsequent p53 activation and CRC cell apoptosis. Blockage of this complexation, though shRNA-mediated knockdown of p53 or AMPKα1, or by AMPKα1 mutilation, inhibited ODE-induced p53 activation and subsequent CRC apoptosis.

It should be noted that inhibition of AMPK via the methods described did not result in total abolition of ODE-mediated cytotoxic effects against CRC cells. Meanwhile, p53 shRNA stable knockdown only inhibited (but not reversed) ODE's actions. Therefore, it is likely that other signalings, proposed by a number of other studies [9–13], could also contribute to the actions by ODE in CRC cells. Thus, it will be interesting to understand the relationship between AMPK signaling and these other possible pathways in mediating ODE's effects. Further studies will also be needed to explore the potential upstream kinases for AMPK activation by ODE.

The other important funding of the study is that *i.p.* injection of ODE at well-tolerated doses significantly inhibited HCT-116 xenograft growth in nude mice. Further, AMPK activation, mTORC1 inhibition and p53 activation were also observed in ODE-treated HCT-116 tumors. These results suggest that ODE could be further investigated as a novel and promising anti-CRC agent.

## MATERIALS AND METHODS

### Ethics

All methods listed in the study were carried out in accordance with the approved guidelines by authors' institutions (Nanjing University of Chinese Medicine, Nanjing Medical University and Jiangsu University).

### Chemicals, reagents and antibodies

*Oldenlandia diffusa* extracts (ODE) were purified and provided by Nanjing University Of Chinese Medicine (Nanjing, China). The caspase-3 specific inhibitor Ac-DEVD-CHO, the caspase-9 inhibitor Ac-LEHD-CHO and the pan caspase inhibitor Ac-VAD-CHO were purchased from Calbiochem (La Jolla, CA). B-cell lymphoma 2 (Bcl-2) antibody (sc-7382), p53 antibody (sc-162), and hypoxia-inducible factor 1-α (HIF-1α) antibody (sc-10790) were obtained from Santa Cruz Biotechnology (Santa Cruz, CA). Antibodies of p-AMPKα1 (Thr172, #2531), AMPKα (#2532), acetyl-CoA Carboxylase (ACC, #3662), p-ACC (Ser79, #3661), p-p53 (Ser15, #9284), p70 S6 Kinase (S6K1 #9202), p-S6K1 (Thr389, #9205), cleaved PARP (#5625), cleaved-caspase-3 (#9664) and β-Tubulin (#2146) were purchased from were obtained from Cell Signaling Technology (Beverly, MA).

### Cell culture

Human CRC cell lines, including HCT-116, DLD-1, HT-29 and Lovo were cultured as described [2]. For all the cell lines, DNA fingerprinting and profiling were performed every 6 months to confirm the origin of the cell line, and to distinguish the cell line from cross-contamination. All cell lines were subjected to mycoplasma and microbial contamination examination. Population doubling time, colony forming efficiency, and morphology under phase contrast were also measured every 6 months under defined conditions to confirm the phonotype of cell line.

### Primary colon cancer cell isolation and culture

Three patients with primary colon cancer administrated at Wuxi People's Hospital of Nanjing Medical University were enrolled in the study. Patient-1, male, 62 years old, Grade I, T2; Patient-2, male, 60 years old, Grade II, T3; Patient-3, male, 56 years old, Grade II, T2; As previously described [2], the surgery-isolated colon cancer specimens were thoroughly washed and minced into small pieces. Samples were then mechanically dissociated and filtered via a 70 μm strainer. Single-cell suspensions of the colon cancer cells were achieved by re-suspending cells in 0.15% (w/v) collagenase I (Sigma) dissolved in DMEM for 1 h, individual cells were pelleted and rinsed twice with DMEM before re-suspending in the cell culture medium as described [2]. The study was approved by the institutional review board (IRB) of all authors' institutions. All clinical investigations were conducted according to the principles expressed in the Declaration of Helsinki. The protocol was approved by authors' institutions. Written-informed consent was obtained from all subjects.

### Methyl thiazol tetrazolium (MTT) assay of cell proliferation

Cell proliferation was assessed via the MTT (Sigma) assay as described [2, 3, 27, 28].

### BrdU incorporation assay of cell proliferation

The proliferation of CRC cells was also estimated via the incorporation of 5-bromo-2′-deoxyuridine (BrdU). Briefly, cells (0.8 ×10^4^/well) were exposed to applied ODE treatment. Afterwards, BrdU (10 μM, Roche Diagnostics, Shanghai, China) was added to the medium, and then the cells were incubated for another 16 h. Next, the cells were fixed, and BrdU incorporation was determined with a cell proliferation enzyme-linked immunosorbent assay (ELISA) kit (Roche Diagnostics) according to the manufacturer's instructions. ELISA OD was utilized as a quantitative measurement of cancer cell proliferation.

### Colonies formation assay

After applied ODE treatment, CRC cells were suspended in 1 mL of DMEM containing 0.25% agar (Sigma). The cell suspension was then added on the top of a pre-solidified 100 mm culture dish. After 10 days of incubation, the number of colonies were fixed, stained and manually counted.

### Trypan blue staining assay of cell death

As described previously [2], following applied treatment, the cell death percentage was determined by counting cells via a hemocytometer, supplemented with trypan blue, which stains the cytoplasm of dead cells [29]. Cell death percentage = the number of trypan blue stained cells/the number of total cells (×100%).

### Assay of caspase-3 activity

As described [30], to test caspase-3 activity, 20 μg of cytosolic protein extracts per sample were mixed with the caspase assay buffer [30] and the caspase-3 substrate Ac-DEVD-AFC (15 μg/mL) (Calbiochem). After 1 h incubation at 37°C, the released AFC was measured through a Shimadzu FC 5300 spectrofluorometer with excitation at 400 nm. The caspase-3 activity of ODE-treatment group was normalized to that of untreated control group.

### Histone-DNA ELISA assay

Cell apoptosis was quantified by Histone-DNA ELISA PLUS kit (Roche Applied Science, Shanghai, China) according to the manufacturer's protocol [3, 27, 28].

### Apoptosis assay by Annexin V fluorescence-activated cell sorting (FACS)

The FACS detecting CRC cell apoptosis was described in our previous study [28]. Propidium iodide (PI) negative and Annexin V positive cells were gated as early apoptotic cells, and PI positive and Annexin V positive cells were gated as late apoptotic cells.

### TUNEL assay of apoptosis

Cell apoptosis was also detected by the TUNEL (Terminal Deoxynucleotidyl Transferase dUTP Nick End Labeling) In Situ Cell Apoptosis Detection Kit (Roche, Shanghai, China), according to the manufacturer's instructions. TUNEL positive nuclei ratio was recorded.

### Western blots

Western blots were performed as previously reported [2, 3, 27, 28]. Blot intensity was quantified by ImageJ software (NIH) after normalization to corresponding loading control.

### Co-immunoprecipitation (Co-IP)

As described [31], after applied treatment, 1000 μg of cell lysates per sample were pre-cleared with 30 μL of protein A/G PLUS-agarose (Santa Cruz) for 1 h. Next, the lysates were centrifuged for 5 min at 4°C in a micro-centrifuge to remove nonspecific aggregates. The supernatant was then rotated overnight with 0.1-0.25 μg of indicated primary antibody (anti-AMPKα1/anti-p53) (Santa Cruz). The protein A/G PLUS-agarose (35 μL/sample) was then added to the supernatants at 4°C for 4 h. Pellets were washed six times with PBS, resuspended in lysis buffer, and then assayed by Western blots.

### Short hairpin RNA (shRNA) and stable cells selection

As described in our previous studies [29], lentiviral particles containing the p53-shRNA-1 (Santa Cruz, sc-29435-V), p53 shRNA-2 (Genechem, Shanghai, China) or AMPKα1 shRNA (Santa Cruz, sc-29673-V) (10 μL/well) were added to cultured CRC cells for 24 h. Afterwards, cell culture medium was replaced with puromycin (0.5 μg/mL)-containing complete medium. The medium was renewed every 2-3 days until resistant stable colonies were formed. Expression of targeted protein (AMPKα1 and p53) was always determined by Western-blots in resulting stable cells. Same amount of scramble control shRNA lentiviral particles (Santa Cruz, sc-108080) were added to control cells.

### AMPK dominant negative mutation

The dominant-negative (dn) mutant of AMPK-α1 (AMPK-α1-T172A) construct was described previously [29]. dn-AMPK-α1 cDNA (0.25 μg/mL) was transfected to CRC cells via the Lipofectamine 2000 protocol [29], stable cells were selected through neomycin (2 μg/mL). Transfection efficiency was again determined by Western blots in resulting stable cells.

### AMPKα1 siRNA knockdown in primary human CRC cells

The AMPK-α1 siRNA(−1) (5′-GCAUAUGCUGC AGGUAGAU-3′), the AMPK-α1 siRNA(−2) (5′-AAGG AAAGTGAAGGTGGGCAA-3′) and the scramble control siRNA were all provided by Dr. Biao Xu's Lab [32]. Transient transfection was performed by the Lipofectamine 2000 reagent (Roche) according to the manufacturer's instructions. Transfection efficiency was determined by Western blots in resulting cells.

### Tumor xenografts

The mice experiments were performed based on the protocol described in our previous studies [4, 29] with minor modifications. The male severe combined immuno-deficient (SCID) *nu/nu* mice were implanted *s.c.* with HCT-116 cells (5×10^6^ per mouse). When the tumors reached the average volume of 200 mm^3^, the mice were divided into three groups: Saline (“Vehicle”), low-dose of ODE group (0.2 g/kg, *i.p.*) and high-dose of ODE group (1.0 g/kg, *i.p.*). ODE was freshly prepared and administered daily for a total of 30 days. Tumor volumes were recorded weekly, calculated via the following formula: π/6 × larger diameter × (smaller diameter)^2^. The mice were maintained as described [29]. All studies were performed in accordance with the standards of ethical treatment approved by the Institutional Animal Care and Use Committee (IACUC) and Association for the Assessment and Accreditation of Laboratory Animal Care (AAALAC). The protocol was approved by authors' institutions (Nanjing University of Chinese Medicine, Nanjing Medical University and Jiangsu University).

### Statistical analysis

Each experiment was repeated a minimum of three times, with similar results obtained each time. Data were presented as mean ± standard deviation (SD). Statistics were analyzed by one-way ANOVA followed by a Scheffe' and Tukey Test by SPSS 15.0 software (SPSS Inc., Chicago, IL). Significance was chosen as *p* < 0.05. The concentrations of agents applied and the treatment durations were chosen based on published literatures and results from our pre-experiments. IC-50 was also calculated by SPSS software [2].
